# Assessing the Impact of Conversational Artificial Intelligence in the Treatment of Stress and Anxiety in Aging Adults: Randomized Controlled Trial

**DOI:** 10.2196/38067

**Published:** 2022-09-23

**Authors:** Morena Danieli, Tommaso Ciulli, Seyed Mahed Mousavi, Giorgia Silvestri, Simone Barbato, Lorenzo Di Natale, Giuseppe Riccardi

**Affiliations:** 1 Signal & Interactive Systems Lab Dipartimento di Ingegneria e Scienze dell'Informazione Università degli Studi di Trento Povo di Trento - Trento Italy; 2 IDEGO - Digital Psychology srl Rome Italy

**Keywords:** mental health care, conversational artificial intelligence, mobile health, mHealth, personal health care agent

## Abstract

**Background:**

While mental health applications are increasingly becoming available for large populations of users, there is a lack of controlled trials on the impacts of such applications. Artificial intelligence (AI)-empowered agents have been evaluated when assisting adults with cognitive impairments; however, few applications are available for aging adults who are still actively working. These adults often have high stress levels related to changes in their work places, and related symptoms eventually affect their quality of life.

**Objective:**

We aimed to evaluate the contribution of TEO (Therapy Empowerment Opportunity), a mobile personal health care agent with conversational AI. TEO promotes mental health and well-being by engaging patients in conversations to recollect the details of events that increased their anxiety and by providing therapeutic exercises and suggestions.

**Methods:**

The study was based on a protocolized intervention for stress and anxiety management. Participants with stress symptoms and mild-to-moderate anxiety received an 8-week cognitive behavioral therapy (CBT) intervention delivered remotely. A group of participants also interacted with the agent TEO. The participants were active workers aged over 55 years. The experimental groups were as follows: group 1, traditional therapy; group 2, traditional therapy and mobile health (mHealth) agent; group 3, mHealth agent; and group 4, no treatment (assigned to a waiting list). Symptoms related to stress (anxiety, physical disease, and depression) were assessed prior to treatment (T1), at the end (T2), and 3 months after treatment (T3), using standardized psychological questionnaires. Moreover, the Patient Health Questionnaire-8 and General Anxiety Disorders-7 scales were administered before the intervention (T1), at mid-term (T2), at the end of the intervention (T3), and after 3 months (T4). At the end of the intervention, participants in groups 1, 2, and 3 filled in a satisfaction questionnaire.

**Results:**

Despite randomization, statistically significant differences between groups were present at T1. Group 4 showed lower levels of anxiety and depression compared with group 1, and lower levels of stress compared with group 2. Comparisons between groups at T2 and T3 did not show significant differences in outcomes. Analyses conducted within groups showed significant differences between times in group 2, with greater improvements in the levels of stress and scores related to overall well-being. A general worsening trend between T2 and T3 was detected in all groups, with a significant increase in stress levels in group 2. Group 2 reported higher levels of perceived usefulness and satisfaction.

**Conclusions:**

No statistically significant differences could be observed between participants who used the mHealth app alone or within the traditional CBT setting. However, the results indicated significant differences within the groups that received treatment and a stable tendency toward improvement, which was limited to individual perceptions of stress-related symptoms.

**Trial Registration:**

ClinicalTrials.gov NCT04809090; https://clinicaltrials.gov/ct2/show/NCT04809090

## Introduction

### Background

The multiplicity of issues related with active aging has been on the agenda of national institutions and health agencies for many years worldwide. The European Union framework directive on health and safety at work (89/391/ EEC) [1] indicates that practicable adjustments to physical and social working environments are necessary to prevent or reduce excessive physical and mental demands on aging workers. Many studies have identified high levels of stress in the workplace as a major factor for developing age-related health risks, including cardiovascular diseases, sickness absence, anxiety, depression, and burnout syndrome [2-5]. As a consequence, several interventions have been implemented and evaluated for the prevention of physical diseases and mental disorders, and the strengthening of older employees, as reported in a recent systematic review [6]. Although this review did not focus only on the older population of workers, it reported some interesting relevant findings for our research. Based on moderate evidence that emerged from the review, cognitive behavioral therapy (CBT) and stress management programs are expected to reduce perceived stress. Nevertheless, the persistence and sustainability of these interventions were insufficient or limited.

Another systematic review analyzed the results of studies providing evidence for digital psychological interventions in the workplace [7]. The authors reviewed digital interventions aimed to address the well-known problem of accessibility of mental health care for the working population in general, due to limited resources in the occupational health sector and to stigma. The adjective “digital” in the reviewed studies stands for interventions whose primary modality of delivery was a website, where participants could access different types of assignments and receive feedback after completing the assignments from a coach or therapist by email, text, or phone call. All the summarized studies were randomized controlled trials (RCTs), but only one study reported data about a mobile app, and no study mentioned artificial intelligence (AI)-empowered treatments.

The demand for accessible and large-scale mental health care support has been previously pointed out [8] and aggravated by the COVID-19 pandemic and its consequences [9,10]. A growing number of studies have indicated that the development of conversational AI systems (also known as chatbots) as applications in the mental health domain can improve access to mental health care support in an easy and inexpensive manner [8,11,12]. Even though traditional in-person therapy sessions remain the most frequent framework for support provision, conversational AI agents have been shown to be an effective alternative regarding various mental disorders, such as stress, anxiety, and depression [8]. In particular, during the COVID-19 pandemic, the problem of accessibility to mental health treatments increased users’ appreciation of remote therapy, thus providing video therapy an opportunity to develop its potential in a world where these kinds of communications represent the new normal [13].

TEO (Therapy Empowerment Opportunity) is a mobile personal health care agent (m-PHA) designed to provide CBT support for the prevention and treatment of stress and anxiety [12]. It has been designed and developed in collaboration with CBT therapists [12]. In the course of the intervention, TEO converses with users through text-based dialogues. From these conversations, TEO recognizes users’ emotional states, beliefs, and personal events, and implements strategies designed by professionals.

### Objective

The observational study discussed in this paper was designed for evaluating the impact of introducing AI technology in the psychological treatment of aging workers presenting a variety of stress symptoms hypothetically related with moderate to high levels of perceived stress in the workplace. The experimental protocol was designed to answer the following questions: (1) whether the use of AI-empowered conversational technologies could contribute to people’s psychological well-being; (2) whether there are differences in terms of symptom reduction between receiving support from an AI-empowered conversational technology and traditional psychotherapy; (3) whether the observed changes are different when comparing a group of people receiving treatment or not receiving it; and (4) whether there are differences compared with a group of people receiving a standard course with a psychologist in a remote setting.

## Methods

### Design

The experimental design included comparison of the presence of several different symptoms, like anxiety and depression, and psychological attitudes, measured by standardized self-assessed psychological questionnaires. We applied these metrics before treatment (T1) and at the end of treatment (T2). An additional measurement was performed 3 months after the end of treatment to longitudinally assess the effects (T3). The self-assessment scales we applied were Symptom Checklist-90-Revised (SCL-90-R), Occupational Stress Indicator (OSI), and Perceived Stress Scale (PSS). SCL-90-R is a self-administered questionnaire that assesses a broad spectrum of psychopathological symptoms like depression, anxiety, psychoticism, and others. OSI is a questionnaire for the evaluation of psychosocial stress in organizations. PSS is a brief questionnaire for the detection of generalized psychological stress. In addition, 2 brief versions of Patient Health Questionnaire-8 (PHQ-8) and General Anxiety Disorders-7 (GAD-7) were administered at the beginning of treatment (T1), after 4 weeks (T2), at the end of treatment (T3), and after 3 months (T4). PHQ-8 is an 8-item questionnaire for assessing and monitoring depression severity [14], while GAD-7 is a short questionnaire for assessing and monitoring generalized anxiety disorders [15].

The treatment involved administering 8 weeks of cognitive behavioral psychotherapy, specifically oriented toward the acquisition of stress management skills. In addition, the experimental design included the possibility of supporting stress management training-CBT with the continuous assistance of an AI-based conversational agent for mental health care (TEO) [12]. The experimental design included 4 groups of subjects as follows: group 1 received traditional psychotherapy from CBT therapists in a remote setting; group 2 received both traditional therapy and the support of the conversational AI agent; group 3 received only the support of the conversational AI agent; and group 4 was the control group not receiving any treatment. Participants assigned to group 4 were also assigned to a waiting list and received treatment at the end of the 8 weeks of the experiment.

IDEGO (Digital Psychology srl, Rome, Italy) carried out the psychometric tests and their data analysis. The experimental design of the RCT, training, and evaluation of the AI algorithms and systems were performed by the University of Trento.

### Ethics Approval

This methodology was approved by the Ethics Committee of the University of Trento within the context of the research activities of the HORIZON2020 CO-ADAPT project, and the experimental protocol has been registered on ClinicalTrials.gov (NCT04809090).

### Recruitment

We collected the data of this study between Spring and Fall 2021, when the third wave of the COVID-19 pandemic was hitting Italy, starting from the Northern regions of the country. The traditional recruitment strategies were inadequate or limited owing to social distancing measures. To overcome these difficulties, we designed new strategies on social media with recruiting campaigns involving engaging posts and graphics. Comparing the usage statistics of the 2 social networks Facebook and Instagram, Facebook provided the highest percentage of users in our target group (21.3% and 11.7% for Facebook and Instagram, respectively) [16]. The campaigns were widespread throughout Italy, with the goal to motivate people to reach our website [17] and enroll in our research. The site included all the information about the research and a form where the users could request to participate. Moreover, the users could ask for more information, resulting in one-to-one interviews to answer all the questions. In order to select eligible participants, several questionnaires and a clinical interview with each subject were conducted. Exclusion criteria were the presence of severe depression (PHQ-8 score ≥20), suicidal thoughts, substance abuse, and mild cognitive impairment (Montreal Cognitive Assessment score <26) [18].

### Participants

**Figure 1:**
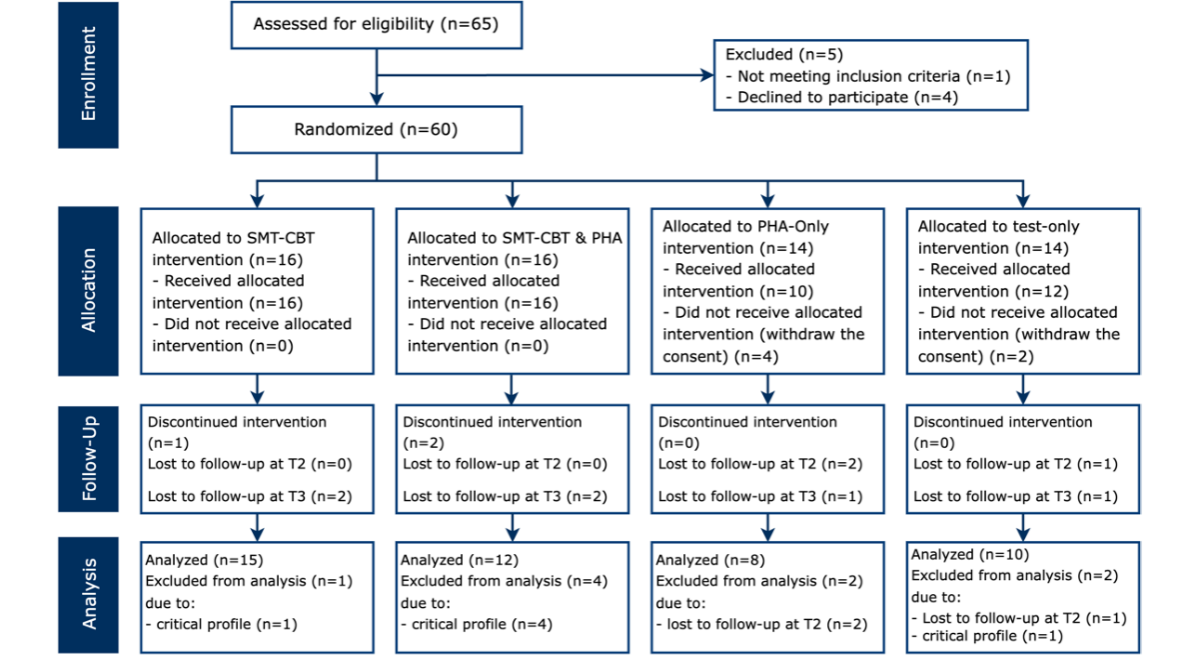
The CONSORT (Consolidated Standards of Reporting Trials) diagram shows the flow of the intervention, the enrollment of participants, their allocation to treatment, their follow-up, and data analysis. PHA: personal health care agent; SMT-CBT: stress management training-cognitive behavioral therapy; T2: end of the treatment; T3: 3 months after the treatment.

The characteristics of the samples are described in Table 1. A total of 65 potential participants were examined, and of these, 60 were recruited. A code was assigned to each participant, and through a random generator of numbers, the selected subjects were distributed into 4 groups. After the assignment, 2 subjects (1 in group 3 and 1 in group 4) showed mental health issues that made it necessary to reassign them to groups 1 and 2 to provide more accurate monitoring, where they could receive psychological support throughout the experiment. Other subjects showed a critical profile during the experiment, and they were directed to a standard psychological support service. Subsequently, these subjects were excluded from the analyses (Figure 1). Only 45 subjects were considered for the analysis. Group 1 included 27% (4/15) men and 73% (11/15) women, with a mean age of 54.08 (SD 4.11; median 54) years. Group 2 included 17% (2/12) men and 83% (10/12) women, with a mean age of 55.17 (SD 3.69; median 55) years. Group 3 included 25% (2/8) men and 75% (6/8) women, with a mean age of 55.63 (SD 4.50; median 55.5) years. Group 4 included 20% (2/10) men and 80% (8/10) women, with a mean age of 57.20 (SD 7.96; median 60) years.

**Table 1:** Sample characteristics (N=45).

Characteristic		Value
Age (years), mean (SD)		55.58 (5.08)
**Gender, n (%)**		
	Male	10 (22)
	Female	35 (78)
**Group, n (%)**		
	Group 1	15 (33)
	Group 2	12 (27)
	Group 3	8 (18)
	Group 4	10 (22)
**Formal education, n (%)**		
	Secondary school	4 (9)
	High school	14 (31)
	Degree	16 (36)
	Master’s degree or PhD	2 (4)
	Other	9 (20)
**Marital status, n (%)**		
	Single	6 (13)
	Cohabiting	2 (5)
	Married	21 (47)
	Separated	15 (33)
	Widower	1 (2)

### TEO

TEO is an m-PHA [19], a type of AI conversational agent, in the form of a mobile app that supports input/output interactions with users via natural language. Many PHAs currently developed for the mental health domain demonstrate limited flexibility of interactions with users, with system-directed interactions and a predefined dialogue flow [11]. As a result, the user has no control over the flow of the conversation and can only follow the system directives throughout the conversation. These limitations lead to shallow conversations and weak user engagement [8].

TEO allows users to share their thoughts and emotions using free-form natural language and engages users in personalized interactions about the events that are specific to each user. TEO can engage users in 2 types of dialogues. For the first type, TEO is designed to facilitate ABC (Activation, Belief, and Consequence) note writing for users. ABC notes are worksheets used by CBT therapists to help their patients in the identification of activating events (A), their beliefs related to the events (B), and the consequences of the events (C). Upon initiatives from a user to share a moment he/she is experiencing, TEO engages the user in dialogues where it asks a controlled set of questions designed by CBT therapists and collects an ABC note from the user in the form of a personal narrative about the event and his/her emotions. For the second type of dialogue (follow-up), TEO notifies the user about the ABC note written the day before and asks the user how he/she feels about the events, whether the issue is resolved, or whether the user is experiencing a different emotion [20]. TEO then tends to engage the user in a short personalized dialogue where it detects the recurrence of emotions and life events the user is experiencing [21], and provides helpful suggestions to ensure a healthier mental state.

Furthermore, TEO benefits from a knowledge base of therapeutic suggestions, recommendations, and exercises, which have been collected from therapists and domain experts. Users receive personalized tips and exercises weekly based on their progress of the therapy intervention. All the interactions with TEO are provided to the therapist weekly prior to the therapy session, so that the therapist can provide necessary support regarding the events and emotions expressed in the recollections and notes.

### Measures

According to the findings by Sullivan and Artino [22] about the power of parametric versus nonparametric tests to detect differences between small-size samples, parametric analysis with repeated measures ANOVA (with a mixed within and between-subjects design) was performed to assess the differences between times (T1, T2, and T3) and groups (group 1, group 2, group 3, and group 4), and their interaction effect related to the results obtained in the PSS, SCL-90-R, and OSI tests. Multiple comparisons were corrected by using Bonferroni adjustment. The same analysis was conducted on PHQ-8 and GAD-7, which were administered before the intervention (T1), at mid-term (T2), at the end of the intervention (T3), and after 3 months (T4) to assess the differences between times (T1, T2, T3, and T4) and groups (group 1, group 2, group 3, and group 4). Regarding the OSI test, only a few scales were considered for the analysis, that is, the ones regarding coping strategies (social support, home-work relationship, task oriented, logic, time, and involvement), mental health, and physical health.

## Results

### PSS and SCL-90-R Results

The results obtained by administering the PSS and SCL-90-R tests are reported in Table 2. For the PSS, Global Severity Index (GSI), Positive Symptom Total (PST), Positive Symptom Distress Index (PSDI), obsessiveness-compulsiveness, interpersonal hypersensitivity, and depression scales/subscales, the assumption of sphericity had not been violated; otherwise, for the hostility and psychoticism subscales, the assumption had been violated (Multimedia Appendix 1 presents the results of the Mauchly test).

For the PSS, lower scores indicate lower stress levels and better well-being. Significant differences within groups between times were found for group 2 between T1 (mean 22.4, standard error [SE] 1.97) and T2 (mean 11.6, SE 2.36) (SE 2.52; *P*<.001), between T2 (mean 11.6, SE 2.36) and T3 (mean 16.6, SE 1.90) (SE 1.80; *P*=.03), and between T1 (mean 22.4, SE 1.97) and T3 (mean 16.6, SE 1.90) (SE 2.01; *P*=.02; Table 2). Further comparisons conducted within times between groups revealed a significant difference between groups at T1 (*F*_3,32_=3.34; *P*=.03; η^2^p=0.24), specifically between group 2 (mean 22.4, SE 1.97) and group 4 (mean 13.88, SE 2.20) (SE 2.95; *P*=.04) at T1.

For the GSI subscale of the SCL-90-R, lower values indicate less psychological distress. Significant differences within groups between times were found for group 2 between T1 (mean 59.4, SE 2.64) and T2 (mean 48.9, SE 3.99) (SE 2.83; *P*=.002; Table 2). Further comparisons conducted within times between groups did not highlight any significant difference.

For the PST subscale, lower scores indicate fewer reported symptoms. Significant differences within groups between times were found for group 2 between T1 (mean 59.7, SE 2.50) and T2 (mean 51.9, SE 3.17) (SE 2.24; *P*=.004; Table 2). Further comparisons conducted within times between groups did not highlight any significant difference.

For the PSDI subscale, lower scores indicate lower intensity of distress. Significant differences within groups between times were found for group 2 between T1 (mean 57, SE 2.51) and T2 (mean 45.1, SE 3.94) (SE 3.37; *P*=.004; Table 2). Further comparisons conducted within times between groups did not highlight any significant difference.

For the obsessiveness-compulsiveness subscale, lower scores indicate less symptomatology. Significant differences within groups between times were found for group 2 between T1 (mean 57.6, SE 2.29) and T2 (mean 47.9, SE 3.53) (SE 3.02; *P*=.009; Table 2). Further comparisons conducted within times between groups did not highlight any significant difference.

For the interpersonal hypersensitivity subscale, lower scores indicate less presence of feelings of inadequacy and inferiority. Significant differences within groups between times were found for group 2 between T1 (mean 54.9, SE 2.05) and T2 (mean 48, SE 2.36) (SE 2.21; *P*=.01; Table 2). Further comparisons conducted within times between groups did not highlight any significant difference.

For the depression subscale, lower scores indicate less depression symptoms. Significant differences within groups between times were found for group 2 between T1 (mean 63.1, SE 3.29) and T2 (mean 51.8, SE 4.79) (SE 3.58; *P*=.01; Table 2). Further comparisons conducted within times between groups did not highlight any significant difference.

For the hostility subscale, lower scores indicate the presence of fewer anger-related personal characteristics. Significant differences within groups between times were found for group 2 between T1 (mean 57.6, SE 4.22) and T2 (mean 45.4, SE 2.21) (SE 4.49; *P*=.03; Table 2). Further comparisons conducted within times between groups did not highlight any significant difference.

For the psychoticism subscale, lower scores indicate less tendency of isolation and less presence of symptoms. Significant differences within groups between times were found for group 2 between T1 (mean 56.7, SE 3.29) and T2 (mean 50.2, SE 3.58) (SE 2.18; *P*=.02; Table 2). Further comparisons conducted within times between groups did not highlight any significant difference.

The results of the somatization, anxiety, phobic anxiety, and paranoid ideation (PAR) subscales are shown in Multimedia Appendix 1.

**Table 2:** Parametric analysis of repeated measures ANOVA for differences between times and groups with regard to the Perceived Stress Scale and Symptom Checklist-90-Revised test.

Scale/subscale and group^a^			Time^b^							*F* (*df*)			*P* value			η^2^p
			T1, mean (SD)		T2, mean (SD)		T3, mean (SD)									
**PSS^c^ score**																
	Group 1	21.17 (6.24)		15.58 (7.81)		16.92 (5.45)		3.22 (2,31)			.053			0.17		
	Group 2	22.40 (5.66)		11.60 (5.85)		16.60 (6.29)		8.95 (2,31)			<.001			0.37		
	Group 3	21.50 (8.17)		14.00 (9.38)		18.67 (7.53)		2.86 (2,31)			.07			0.16		
	Group 4	13.87 (5.19)		14.63 (7.15)		15.13 (5.25)		0.17 (2,31)			.85			0.01		
**GSI^d^**																
	Group 1	58.42 (11.47)		56.33 (18.73)		53.17 (12.58)		2.16 (2,31)			.13			0.12		
	Group 2	59.40 (5.72)		48.90 (7.36)		54.70 (11.31)		6.77 (2,31)			.004			0.30		
	Group 3	54.67 (6.89)		48.83 (6.59)		50.50 (9.89)		1.44 (2,31)			.25			0.09		
	Group 4	53.25 (6.07)		49.88 (8.74)		51.50 (9.47)		0.57 (2,31)			.57			0.04		
**PST^e^ score**																
	Group 1	57.00 (9.41)		54.92 (11.99)		53.50 (11.97)		1.10 (2,31)			.35			0.07		
	Group 2	59.70 (6.38)		51.90 (9.21)		55.00 (12.24)		5.97 (2,31)			.006			0.28		
	Group 3	56.00 (9.10)		48.67 (8.04)		50.50 (9.94)		3.14 (2,31)			.057			0.17		
	Group 4	56.88 (5.99)		51.63 (8.91)		53.13 (9.75)		2.14 (2,31)			.14			0.12		
**PSDI^f^**																
	Group 1	57.08 (10.25)		53.00 (16.83)		51.75 (9.96)		1.90 (2,31)			.17			0.11		
	Group 2	57.00 (8.49)		45.10 (6.59)		51.60 (9.57)		6.49 (2,31)			.004			0.30		
	Group 3	53.00 (4.73)		53.00 (14.99)		49.33 (9.48)		0.33 (2,31)			.72			0.02		
	Group 4	48.75 (3.88)		46.88 (7.00)		48.50 (8.19)		0.12 (2,31)			.89			0.01		
**Somatization score**																
	Group 1	57.25 (15.02)		53.83 (21.91)		52.83 (15.12)		1.15 (2,31)			.33			0.07		
	Group 2	55.90 (10.96)		47.30 (7.43)		49.60 (8.75)		2.99 (2,31)			.06			0.16		
	Group 3	49.67 (6.65)		45.83 (6.37)		42.83 (3.87)		1.40 (2,31)			.26			0.08		
	Group 4	51.38 (8.78)		50.50 (8.33)		50.88 (8.63)		0.02 (2,31)			.98			0.00		
**Obsessiveness-compulsiveness score**																
	Group 1	56.83 (8.62)		56.00 (15.58)		54.75 (11.34)		0.34 (2,31)			.72			0.02		
	Group 2	57.60 (7.04)		47.90 (7.91)		53.50 (11.08)		4.99 (2,31)			.01			0.24		
	Group 3	55.83 (7.63)		51.67 (7.03)		50.67 (11.24)		1.13 (2,31)			.34			0.07		
	Group 4	53.38 (4.21)		51.00 (8.47)		51.88 (8.06)		0.26 (2,31)			.78			0.02		
**Interpersonal sensitivity score**																
	Group 1	52.25 (6.11)		48.67 (7.23)		50.17 (10.47)		1.53 (2,31)			.23			0.09		
	Group 2	54.90 (6.33)		48.00 (6.60)		51.60 (11.46)		4.71 (2,31)			.02			0.23		
	Group 3	53.67 (8.43)		45.83 (5.31)		52.33 (9.07)		3.87 (2,31)			.03			0.20		
	Group 4	53.88 (5.57)		49.50 (9.84)		52.63 (15.90)		1.55 (2,31)			.23			0.09		
**Depression score**																
	Group 1	59.25 (12.13)		57.67 (22.76)		55.67 (11.26)		0.72 (2,31)			.50			0.04		
	Group 2	63.10 (9.79)		51.80 (9.46)		56.60 (13.13)		5.34 (2,31)			.01			0.26		
	Group 3	55.33 (9.27)		48.17 (7.63)		54.00 (13.23)		1.19 (2,31)			.32			0.07		
	Group 4	54.63 (8.86)		52.13 (8.86)		51.88 (9.03)		0.36 (2,31)			.70			0.02		
**Anxiety score**																
	Group 1	56.92 (13.59)		57.50 (23.62)		53.50 (10.37)		0.89 (2,31)			.42			0.05		
	Group 2	56.50 (11.57)		47.80 (5.45)		54.10 (9.61)		2.27 (2,31)			.12			0.13		
	Group 3	55.83 (6.94)		51.33 (8.57)		52.67 (11.29)		0.48 (2,31)			.62			0.03		
	Group 4	50.75 (5.31)		46.00 (6.16)		48.38 (6.78)		0.59 (2,31)			.56			0.04		
**Hostility score**																
	Group 1	47.50 (17.58)		48.33 (7.05)		47.33 (8.69)		0.12 (2,31)			.89			0.01		
	Group 2	57.60 (14.52)		45.40 (3.69)		48.80 (7.90)		4.12 (2,31)			.03			0.21		
	Group 3	53.50 (7.99)		49.83 (9.33)		52.00 (8.90)		0.39 (2,31)			.68			0.03		
	Group 4	51.75 (3.28)		46.88 (8.08)		48.63 (6.50)		0.60 (2,31)			.56			0.04		
**Phobic anxiety score**																
	Group 1	51.58 (11.02)		57.67 (21.64)		50.50 (9.56)		2.25 (2,31)			.12			0.13		
	Group 2	50.00 (16.67)		50.60 (6.02)		56.30 (11.38)		2.38 (2,31)			.11			0.13		
	Group 3	48.83 (5.14)		45.67 (3.14)		44.83 (1.60)		0.34 (2,31)			.72			0.02		
	Group 4	48.50 (5.43)		48.38 (5.34)		50.00 (10.92)		0.13 (2,31)			.88			0.01		
**Paranoid ideation score**																
	Group 1	59.92 (10.98)		52.33 (11.76)		53.92 (13.07)		6.58 (2,31)			.004			0.30		
	Group 2	54.20 (9.66)		49.50 (7.82)		54.90 (15.42)		3.49 (2,31)			.04			0.18		
	Group 3	56.50 (10.77)		46.83 (4.96)		48.83 (7.14)		5.35 (2,31)			.01			0.26		
	Group 4	52.25 (8.80)		45.25 (7.44)		50.63 (11.41)		4.57 (2,31)			.02			0.23		
**Psychoticism score**																
	Group 1	56.50 (12.75)		54.67 (15.20)		49.67 (8.79)		3.17 (2,31)			.06			0.17		
	Group 2	56.70 (9.56)		50.20 (8.87)		57.20 (12.64)		4.41 (2,31)			.02			0.22		
	Group 3	48.33 (6.15)		48.17 (6.01)		48.33 (7.47)		0.00 (2,31)			>.99			0.00		
	Group 4	52.63 (9.74)		52.88 (9.75)		52.38 (12.42)		0.01 (2,31)			.99			0.00		

^a^Group 1 received only traditional therapy; group 2 received both traditional therapy and the support of a conversational artificial intelligence agent; group 3 received only the support of a conversational artificial intelligence agent; and group 4 did not receive any treatment (control group).

^b^T1 indicates before treatment, T2 indicates at the end of treatment, and T3 indicates 3 months after the end of treatment.

^c^PSS: Perceived Stress Scale.

^d^GSI: Global Severity Index.

^e^PST: Positive Symptom Total.

^f^PSDI: Positive Symptom Distress Index.

### OSI Results

The main results of the OSI are reported in Table 3. For the task-oriented, logic, mental health, and physical health subscales, the assumption of sphericity had not been violated (Multimedia Appendix 1 presents the results of the Mauchly test).

For the task-oriented subscale, lower scores indicate criticality. Significant differences within groups between times were found for group 2 between T1 (mean 5.2, SE 0.56) and T2 (mean 6.9, SE 0.55) (SE 0.62; *P*=.04; Table 3). Further comparisons conducted within times between groups did not highlight any significant difference.

**Table 3:** Parametric analysis of repeated measures ANOVA for differences between times and groups with regard to the Occupational Stress Inventory.

Subscale and group^a^		Time^b^				*F* (*df*)		*P* value		η^2^p
		T1, mean (SD)	T2, mean (SD)	T3, mean (SD)						
**Social support score**										
	Group 1	7.13 (1.73)	6.50 (2.62)	5.88 (2.85)	1.57 (2,18)		.24		0.15	
	Group 2	5.20 (2.15)	6.10 (1.85)	6.10 (2.77)	1.68 (2,18)		.21		0.16	
	Group 3	5.00 (3.00)	5.00 (1.73)	5.33 (3.79)	0.07 (2,18)		.93		0.01	
	Group 4	7.00 (2.83)	7.50 (2.12)	7.50 (0.71)	0.10 (2,18)		.90		0.01	
**Task-oriented score**										
	Group 1	5.25 (1.98)	5.88 (2.30)	5.50 (3.12)	0.40 (2,18)		.67		0.04	
	Group 2	5.20 (1.55)	6.90 (1.45)	6.70 (2.50)	3.97 (2,18)		.04		0.31	
	Group 3	6.33 (1.16)	5.33 (0.58)	4.33 (3.06)	1.16 (2,18)		.34		0.11	
	Group 4	7.00 (2.83)	6.00 (0.00)	7.00 (1.41)	0.33 (2,18)		.72		0.04	
**Home-work relationship score**										
	Group 1	6.63 (1.69)	7.00 (1.07)	5.75 (1.58)	2.12 (2,18)		.15		0.19	
	Group 2	5.80 (1.32)	6.30 (1.64)	7.00 (1.33)	1.93 (2,18)		.17		0.18	
	Group 3	6.33 (2.08)	5.00 (1.73)	6.67 (2.52)	1.56 (2,18)		.24		0.15	
	Group 4	8.00 (1.41)	7.50 (0.71)	8.50 (0.71)	0.33 (2,18)		.72		0.04	
**Logic score**										
	Group 1	3.88 (1.64)	5.63 (2.50)	4.63 (2.33)	5.48 (2,18)		.01		0.38	
	Group 2	5.20 (1.81)	5.20 (1.75)	5.10 (2.08)	0.02 (2,18)		.99		0.00	
	Group 3	6.00 (1.00)	5.00 (0.00)	3.33 (2.52)	2.50 (2,18)		.11		0.22	
	Group 4	6.00 (1.41)	6.00 (2.83)	6.00 (0.00)	0.00 (2,18)		>.99		0.00	
**Time score**										
	Group 1	4.63 (2.13)	4.63 (2.26)	4.25 (1.75)	0.15 (2,18)		.86		0.02	
	Group 2	5.00 (2.00)	6.00 (1.83)	5.70 (2.45)	1.62 (2,18)		.23		0.15	
	Group 3	4.33 (1.16)	5.33 (2.08)	4.67 (1.53)	0.57 (2,18)		.57		0.06	
	Group 4	6.00 (4.24)	7.00 (2.83)	7.00 (1.41)	0.33 (2,18)		.72		0.04	
**Involvement score**										
	Group 1	5.25 (1.58)	6.50 (1.31)	5.13 (1.81)	1.79 (2,18)		.20		0.17	
	Group 2	5.70 (2.31)	6.70 (1.57)	6.00 (2.21)	1.16 (2,18)		.34		0.11	
	Group 3	6.33 (1.16)	6.00 (1.00)	6.00 (2.65)	0.06 (2,18)		.95		0.01	
	Group 4	7.50 (2.12)	6.50 (4.95)	7.50 (0.71)	0.26 (2,18)		.77		0.03	
**Mental health score**										
	Group 1	6.00 (2.98)	5.13 (2.59)	5.75 (1.67)	0.70 (2,18)		.51		0.07	
	Group 2	4.50 (2.01)	3.00 (2.00)	5.10 (3.32)	6.70 (2,18)		.007		0.43	
	Group 3	4.33 (3.06)	3.33 (2.52)	5.00 (3.61)	1.27 (2,18)		.31		0.12	
	Group 4	2.00 (1.41)	2.00 (1.41)	1.00 (0.00)	0.42 (2,18)		.67		0.04	
**Physical health score**										
	Group 1	6.75 (2.87)	5.75 (2.05)	5.75 (1.28)	0.90 (2,18)		.43		0.09	
	Group 2	7.60 (1.90)	5.70 (2.00)	5.50 (2.99)	4.17 (2,18)		.03		0.32	
	Group 3	6.00 (3.00)	4.67 (2.52)	5.33 (4.16)	0.61 (2,18)		.55		0.06	
	Group 4	4.50 (2.12)	3.00 (1.41)	7.50 (3.54)	2.70 (2,18)		.09		0.23	

^a^Group 1 received only traditional therapy; group 2 received both traditional therapy and the support of a conversational artificial intelligence agent; group 3 received only the support of a conversational artificial intelligence agent; and group 4 did not receive any treatment (control group).

^b^T1 indicates before treatment, T2 indicates at the end of treatment, and T3 indicates 3 months after the end of treatment.

For the logic subscale, lower scores indicate criticality. Significant differences within groups between times were found for group 1 between T1 (mean 3.88, SE 0.59) and T2 (mean 5.63, SE 0.72) (SE 0.52; *P*=.01; Table 3). Further comparisons conducted within times between groups did not highlight any significant difference.

For the mental health subscale, lower scores indicate a higher level of mental well-being. Significant differences within groups between times were found for group 2 between T2 (mean 3.0, SE 0.72) and T3 (mean 5.1, SE 0.87) (SE 0.56; *P*=.004; Table 3). Further comparisons conducted within times between groups did not highlight any significant difference.

For the physical health subscale, lower scores indicate a higher level of physical well-being. Significant differences within groups between times were found for group 2 between T1 (mean 7.6, SE 0.77) and T2 (mean 5.7, SE 0.65) (SE 0.66; *P*=.03; Table 3). Further comparisons conducted within times between groups did not highlight any significant difference.

The results of the social support, home-work relationship, time, and involvement subscales are shown in Multimedia Appendix 1.

### PHQ-8 and GAD-7 Results

The main results of PHQ-8 and GAD-7 are reported in Table 4. For the PHQ-8 test, lower scores indicate lower levels of depression. The only significant difference found was the one between groups at T1 (*F*_3,31_=3.85; *P*=.02; η^2^p=0.27), specifically between group 1 (mean 9.42, SE 1.16) and group 4 (mean 3.43, SE 1.51) (SE 1.91; *P*=.02).

For the GAD-7 test, lower scores indicate lower levels of generalized anxiety. Significant differences within groups between times were found for group 1 between T1 (mean 9.5, SE 1.21) and T4 (mean 4.83, SE 1.1) (SE 1.24; *P*=.004; Table 4). Furthermore, comparisons conducted within times between groups revealed significant differences between groups at T1 (*F*_3,31_=3.53; *P*=.03; η^2^p=0.25), specifically between group 1 (mean 9.5, SE 1.21) and group 4 (mean 3.14, SE 1.58) (SE 1.99, *P*=.02). 

The PSS, SCL-90-R, OSI, PHQ-8, and GAD-7 results of the interaction effects between time and group can be found in Multimedia Appendix 1.

**Table 4:** Parametric analysis of repeated measures ANOVA for differences between times and groups with regard to Patient Health Questionnaire-8 and General Anxiety Disorders-7.

Scale/group^a^			Time^b^									*F* (*df*)			*P* value			η^2^p
			T1, mean (SD)		T2, mean (SD)		T3, mean (SD)		T4, mean (SD)									
**PHQ-8^c^ score**																		
	Group 1	9.42 (4.89)		7.50 (4.76)		7.58 (5.58)		5.83 (4.95)		3.17 (3,29)			.04			0.25		
	Group 2	6.30 (4.72)		6.70 (5.14)		5.60 (5.10)		6.70 (5.74)		0.52 (3,29)			.67			0.05		
	Group 3	4.83 (2.14)		5.50 (5.36)		5.17 (4.02)		4.50 (3.56)		0.10 (3,29)			.96			0.01		
	Group 4	3.43 (1.40)		5.00 (2.71)		5.43 (2.64)		3.14 (1.46)		0.90 (3,29)			.45			0.09		
**GAD-7^d^ score**																		
	Group 1	9.50 (5.23)		8.00 (6.67)		7.08 (5.14)		4.83 (4.02)		4.45 (3,29)			.01			0.32		
	Group 2	7.10 (3.64)		5.70 (2.45)		4.50 (2.76)		5.80 (3.80)		1.19 (3,29)			.33			0.11		
	Group 3	6.00 (4.43)		5.67 (6.25)		5.00 (4.86)		4.50 (4.76)		0.25 (3,29)			.86			0.03		
	Group 4	3.14 (2.04)		4.43 (1.72)		5.14 (1.77)		2.57 (2.23)		1.16 (3,29)			.34			0.11		

^a^Group 1 received only traditional therapy; group 2 received both traditional therapy and the support of a conversational artificial intelligence agent; group 3 received only the support of a conversational artificial intelligence agent; and group 4 did not receive any treatment (control group).

^b^T1 indicates the beginning of treatment, T2 indicates after 4 weeks, T3 indicates at the end of treatment, and T4 indicates after 3 months.

^c^PHQ-8: Patient Health Questionnaire-8.

^d^GAD-7: General Anxiety Disorders-7.

### Participant Feedback

At the end of treatment, feedback was collected from all the participants through the administration of a satisfaction questionnaire conceived for this study. For each item of the questionnaire, users were asked to indicate their degree of agreement on a 5-point Likert scale, from 1 (strongly disagree) to 5 (strongly agree). To assess satisfaction across all groups, 1 item of the questionnaire asked the users if they were satisfied overall with the received treatment. In the same way, to assess usefulness, they were asked if they felt that the treatment was useful. General results of satisfaction and perceived usefulness are shown in Table 5.

In addition to the general questions available for all groups, some specific questions were asked to assess the experience of the participants who could interact with TEO (ie, groups 2 and 3), focusing on the participants’ experiences with the conversational agent. The results are shown in Table 6. “Easy to use” was used to refer to the ease of interaction with TEO, and “usefulness” was used to refer to the perceived usefulness of the app. “Personal usage” was intended to investigate if, in case the conversational agent was available on app stores (iOS or Android), the users would use it (using the question “If TEO was available on the Android/iOS store, would you use/download it for your personal use?”). Statistical analysis with one-way ANOVA was conducted to assess whether there were significant differences between groups for the above variables. No significance was detected. Specific results are reported in Multimedia Appendix 1.

**Table 5:** Satisfaction and perceived utility of the treatment.

Variable	Group 1^a^	Group 2^b^	Group 3^c^
Satisfaction score, mean (SD)	4.21 (0.89)	4.54 (0.66)	4.29 (0.76)
Usefulness score, mean (SD)	4.21 (0.89)	4.69 (0.63)	4.29 (0.76)

^a^Group 1 received only traditional therapy.

^b^Group 2 received both traditional therapy and the support of a conversational artificial intelligence agent.

^c^Group 3 received only the support of a conversational artificial intelligence agent.

**Table 6:** Participants’ self-assessments of mobile personal health care agent interactions.

Variable	Group 2^a,b^	Group 3^a,c^
Easy to use score, mean (SD)	3.62 (1.04)	3.43 (1.40)
Usefulness score, mean (SD)	3.38 (0.87)	3.29 (1.38)
Personal usage score, mean (SD)	3.77 (1.09)	3.14 (1.77)

^a^All values reported represent the average of the group scores.

^b^Group 2 received both traditional therapy and the support of a conversational artificial intelligence agent.

^c^Group 3 received only the support of a conversational artificial intelligence agent.

## Discussion

### Principal Findings

Given the small number of subjects per group, the results concerning the differences between groups and between times within each group are discussed. The statistical analysis seemed to show significant differences between groups as follows: at T1, group 2 and group 4 differed in terms of the PSS, and group 1 and group 4 differed in terms of the GAD-7 and PHQ-8 scales. More specifically, group 2 reported higher levels of stress than group 4, group 1 reported higher levels of anxiety than group 4, and group 1 reported higher levels of depression than group 4. Although randomization of the groups was performed (explained in the Participants subsection in the Methods section), the differences at T1 for the GAD-7, PSS, and PHQ-8 scales could be due to a reduced sample size and a nonuniform distribution in the groups of subjects from different geographical zones of Italy as shown in Table 7. Overall, there was a worsening trend in almost all scales across all groups (Tables 2 and 3) between T2 and T3, although it did not appear to be significant. When we compared the interviews with some subjects and the Italian COVID-19 epidemiological statistics, we could observe an increase in COVID-19 positive cases and a general concern arising from the Delta variant of the virus in conjunction when the T3 statistics were collected from the participants after several months of general stability. This may be the reason for the overall deterioration observed from T2 to T3.

**Table 7:** Distribution of the sample according to the zones of Italy (North, Center, and South).

Zone	Group^a^, n (%)					Total (N=45), n (%)
	Group 1 (n=15)	Group 2 (n=12)	Group 3 (n=8)	Group 4 (n=10)		
North	1 (6.7)	4 (33.3)	2 (25.0)	0 (0.0)	7 (15.6)	
Center	12 (80.0)	6 (50.0)	6 (75.0)	9 (90.0)	33 (73.3)	
South	2 (13.3)	2 (16.7)	0 (0.0)	1 (10.0)	5 (11.1)	

^a^Group 1 received only traditional therapy; group 2 received both traditional therapy and the support of a conversational artificial intelligence agent; group 3 received only the support of a conversational artificial intelligence agent; and group 4 did not receive any treatment (control group).

Analysis conducted separately within each group showed that there were many significant differences between times in group 2. Altogether, group 2 seemed to show improvements in the PSS, GSI, PST, PSDI, obsessiveness-compulsiveness, interpersonal hypersensitivity, depression, hostility, and psychoticism scores (Table 2), as well as the task-oriented, mental health, and physical health scores (Table 3). Despite significant improvements in group 2, for the PSS, there was significant worsening between T2 and T3, and there was an increase in stress at T3, although it was lower than that at T1. There was also worsening of psychological symptoms generally related to stress for the mental health scale of the OSI questionnaire (see Table 3 above). Despite significant worsening of the PSS and mental health (OSI) scores from T2 to T3, which generally detect similar symptoms of stress, it emerged that the physical health (OSI) score improved. This could indicate that subjects in group 2 were more susceptible to sudden changes, that is, increased cases of the contagious disease at T3 in their residential areas (in particular in Northern Italy) that could have increased psychological stress. However, the results for other scales suggest that the participants living in that geographical area could sufficiently cope with increased COVID-19–related worries, without developing higher levels of stress-related physical symptoms.

Group 3 reported significant improvements in the scores of the interpersonal hypersensitivity and PAR scales of the SCL-90-R questionnaire between T2 and T3 (see Table 2). Group 1 reported improved PAR (Table 2), logic (Table 3), and GAD-7 (Table 4) scores between times. Furthermore, group 4 showed a significant improvement in the PAR score between times (Table 2). In group 3, several individuals withdrew their consent to participate. Among them, 2 withdrew their consent owing to organizational complications that emerged and 2 withdrew their consent owing to very high expectations of the conversational AI agent that were not maintained. They judged that it was a waste of time to participate in this research compared with the perceived benefits.

The feedback questionnaires administered to understand the users’ experiences showed that group 2 experienced higher levels of satisfaction and perceived the usefulness of the received treatment more than the other groups (ie, psychological support and use of the mobile health [mHealth] agent), as shown in Table 5. Moreover, comparing groups 2 and 3, participants in group 2 showed a greater ease of interaction with TEO, and they found it more useful than those in group 3 (ie, the group that interacted with the conversational AI agent without human psychological support). Indeed, the “personal usage” scores revealed a greater inclination of group 2 participants to use TEO.

A few specific questions were administered per group to explore some expectations. In group 1, the aim was to understand whether users would accept or find useful the use of a mHealth app together with traditional treatment. The results showed a score of 3.07. In group 3, the aim was to understand whether users would accept or find useful the use of a mHealth app together with traditional treatment. The results showed a score of 4.57. Overall, positive expectations related to combining traditional treatment with a mHealth app were found, considering the fact that participants in group 3, who used the app, had higher expectations. In group 2, the aim was to understand not the expectations but how effectively, for the subjects, the use of the mHealth app facilitated traditional treatment. The results showed a score of 3.93.

Altogether, these results suggest that a psychological treatment, characterized by the presence of human contact, along with a conversational mHealth agent would improve the impact of treatment in terms of satisfaction and usefulness.

A further aspect to be considered in the evaluation of these results is that this experiment was performed during the third wave of the COVID-19 pandemic in Italy, as mentioned in the Methods section. Our results indicate that although this event had an impact on the levels of stress and on the general psychological well-being of the participants, the observed and perceived improvements were maintained over time in terms of the reduction of physical stress–related symptoms.

### Limitations

Following the recruitment process, the number of active participants involved in this study was small, and this may weaken the inferences and conclusions. Besides, although the recruitment campaign was conducted through social media platforms to reach out to all Italian regions, the majority of our participants were from the center of Italy. The participants were mainly women, and the fact that women tend to seek psychological help more often than men has been studied previously [23,24]. Nevertheless, the observed gender predominance weakens the generalization of the drawn inferences for both genders.

### Conclusions

The aim of this study was to evaluate the possible improvements related to the introduction of an AI-based mHealth app in psychological interventions aiming to reduce stress-related physical and psychological symptoms in aging workers. We administered different standard psychological tests to measure the levels of perceived stress, generalized anxiety, and depression, along with other psychological dimensions. We could not observe statistically significant differences between the participants who used the mHealth app alone and those who used it within the traditional setting of psychological treatment. On the contrary, we could observe significant within-group differences, with improvements in subjects who received treatment. Moreover, we observed greater levels of satisfaction and subjective perception of usefulness in participants who were supported by a human therapist as well as the mHealth conversational agent.

## Appendix

Multimedia Appendix 1 Supplementary results.

Multimedia Appendix 2 CONSORT-eHEALTH checklist (V 1.6.1).

## Abbreviations

ABC: Activation, Belief, and Consequence
AI: artificial intelligence
CBT: cognitive behavioral therapy
GAD-7: General Anxiety Disorders-7
GSI: Global Severity Index
mHealth: mobile health
m-PHA: mobile personal health care agent
OSI: Occupational Stress Indicator
PAR: paranoid ideation
PHQ-8: Patient Health Questionnaire-8
PSDI: Positive Symptom Distress Index
PSS: Perceived Stress Scale
PST: Positive Symptom Total
RCT: randomized controlled trial
SCL-90-R: Symptom Checklist 90 Revised
SE: standard error
TEO: Therapy Empowerment Opportunity
