# Supplementary material for: Assessing the Impact of Conversational Artificial Intelligence in the Treatment of Stress and Anxiety in Aging Adults: Randomized Controlled Trial
Source: JMIR Ment Health. 2022 Sep 23;9(9):e38067. doi: 10.2196/38067 (PMC9547337; doi:10.2196/38067)
Supplement: Multimedia Appendix 1 [file mental_v9i9e38067_app1.docx]

## Multimedia Appendix 1

### PSS and Scl-90-R results

For the PSS test, Mauchy’s test had χ^2^(2)=5.63, p=.06. For this scale there was a significant interaction effect between the time and the belonging group, F(6,64)=2.42, p=.036, η2p=0.19; contrast test was performed, showing significant interactions of the groups between T1 and T2, F(3,32)=3.22, p=.036, η2p=0.23. There was also a significant main effect of the time on the levels of the variable under consideration, F(2,64)=12.43, p<.001, η2p=0.28. Contrasts revealed that levels at T1 were significantly higher than levels at T2, F(1,32)=17.82, p<.001, η2p=0.36. Conversely, levels at T2 were significantly lower than T3, F(1,32)=8.57, p=.006, η2p=0.21.

For the GSI subscale of the SCL-90-R, Mauchy’s test  χ^2^(2)=0.93, p=.63. For this subscale the results did not show any significant effect of the interaction between time and belonging group, F(6,64)=1.23, p=.301, η2p= 0,10; whereas, significant effect of the time on the considered variable was found, F(2,64)=2.36, p=.003, η2p= 0,17. Repeated contrasts were performed to better understand this interaction, showing significant interaction between T1 and T2, F(1,32)=12.53, p=.001, η2p=0,28.

For the PST subscale, Mauchy’s test  χ^2^(2)=1.62, p=.444. For this subscale, the results did not show any significant effect of the interaction between time and belonging group, F(6,64)=0.73, p=.625, η2p= 0,6; whereas, significant effect of the time on the considered variable was found, F(2,64)=10.98, p<.001, η2p= 0,26. Similarly, Repeated contrasts were performed to better understand this interaction, showing significant interaction between T1 and T2, F(1,32)=21.26, p<.001, η2p=0,4.

For the PSDI subscale, Mauchy’s test χ^2^(2)=4.29, p=.118. For this subscale, no significant effects were found neither for time, F(2, 64)=2.67, p=.077, η2p= 0,08, nor for the interaction with the group belonging, F(6, 64)=1.06, p=.396, η2p= 0,09. In spite of these results, contrasts revealed a significant interaction between T1 and T2, F(1,32)=5.92, p=.02, η2p=0.156.

For the SOM subscale, Mauchy’s test  χ^2^(2)=1.64, p=.440. For the O-C subscale, Mauchy’s test  χ^2^(2)=0.48, p=.787. For this subscale, the results did not show any significant effect of the interaction between time and belonging group, F(6,64)=1.08, p=.387, η2p= 0,9; whereas, significant effect of the time on the considered variable was found, F(2,64)=3.89, p=.026, η2p= 0,11. Repeated contrasts were performed to better understand this interaction, showing significant interaction between T1 and T2, F(1,32)=6.73, p=.014, η2p=0,17.

For the I-S subscale Mauchy’s test  χ^2^(2)=4.85, p=.874. For this subscale the results did not show any significant effect of the interaction between time and belonging group, F(6,64)=0.33, p=.922, η2p=0,3; whereas, there was a significant main effect of the time on the levels of the variable under consideration, F(2,64)=7.05, p=.002, η2p=0.18. Contrasts revealed that levels at T1 were significantly higher than levels at T2, F(1,32)=22.12, p<.001, η2p=0.41. Conversely, levels at T2 were significantly lower than T3, F(1,32)=4.84, p=.035, η2p=0.13.

For the DEP subscale, Mauchy’s test had  χ^2^(2)=1.68, *p*=.432. For this subscale, the results did not show any significant effect of the interaction between time and belonging group, F(6,64)=0.96, p=.458, η2p= 0,8; whereas, significant effect of the time on the considered variable was found, F(2,64)=4.23, p=.019, η2p= 0,12. Repeated contrasts were performed to better understand this interaction, showing significant interaction between T1 and T2, F(1,32)=8.37, p=.007, η2p=0,21.

For the HOS subscale, with Mauchy’s test χ^2^(2)=18.69, *p*<.001 indicated that the sphericity had been violated, so degrees of freedom were corrected using Greenhouse-Geisser estimates of sphericity (ε = .69). For this subscale, no significant effects were found, neither for time, F(1.38, 44.05)=2.74, p=.093, η2p= 0,08, nor for the interaction with the group belonging, F(4.13, 44.05)=1.04, p=.398, η2p= 0,09. Despite this, contrasts displayed a significance at p=.05 between T1 and T2, F(1,32)=4.13, p=.05, η2p=0.11.

For the PSY, no significant effects were found, neither for time, F(1.58, 50.52)=0.90, p=.391, η2p= 0,03, nor for the interaction with the group belonging, F(4.74, 50.52)=1.80, p=.133, η2p= 0,14.

For the PAR subscale Mauchy’s test had  χ^2^(2)=4.16, *p*=.125. For the PSY subscale with Mauchy’s test had χ^2^(2)=9.63, *p*=.008  indicated that the sphericity had been violated, so degrees of freedom were corrected using Greenhouse-Geisser estimates of sphericity (ε = .79).

For the ANX subscale, Mauchy’s test (χ^2^(2)=3.05, *p*=.218). PHOB subscale, with Mauchy's test(χ^2^(2)=6.98, *p*=.03 indicated that the sphericity had been violated, so degrees of freedom were corrected using Greenhouse-Geisser estimates of sphericity (ε = .83)

For SOM subscale, results did not show any significant effect of the interaction between time and belonging group, F(6,64)=0.63, p=.705,  η^2^p= 0,06; whereas, significant effect of the time on the considered variable was found, F(2,64)=1.02, p=.023, η^2^p= 0,11. Repeated contrasts were performed to better understand this interaction, showing significant interaction between T1 and T2, F(1,32)=4.54, p=.041, η^2^p= 0,12. For this subscale, lower scores indicate less physical impact. Further comparisons did not show any significance neither within groups through time, nor between groups.

For PAR subscale, results did not show any significant effect of the interaction between time and belonging group, F(6,64)=0.95, *p*=.463, η^2^p= 0,08; whereas, there was a significant main effect of the time on the levels of the variable under consideration, F(2,64)=12.52, *p*<.001, η^2^p=0.28. Contrasts revealed that levels at T1 were significantly higher than levels at T2, F(1,32)=34.72, *p*<.001, η^2^p=0.52. Conversely, levels at T2 were significantly lower than T3, F(1,32)=6.49, *p*=.016, η^2^p=0.17. For this scale, lower scores indicate less paranoid thoughts. More specifically, significant differences within groups between times were found for more than one group, as shown in Table 2: G1 presented a significant difference between T1 (Mean=59.92, SE=2.93) and T2(Mean=52.33, SE=2.59) (SE=2.06, *p*=.003); G3 presented a significant difference between T1 (Mean=56.5, SE=4.14) and T2 (Mean=46.43, SE=3.68)(SE=2.91, *p*=.007); G4, between T1 (Mean=52.25, SE=3.58) and T2 (Mean=45.25, SE=3.18)(SE=2.52, *p*=.027). Further comparisons conducted within times between groups did not highlight any significant difference.

For the following subscales, no significant effects were found at any level; for ANX subscale,  neither for time, F(2, 64)=2.31, *p*=.108, η^2^p=0,07, nor for the interaction with the group belonging, F(6, 64)=0.85, *p*=.538, η^2^p= 0,07, as well as for the PHOB subscale, no significance, neither for time, F(1.67, 52.27)=0.08, *p*=.341, η^2^p=0,00, nor for the interaction with the group, F(4.99, 53.27)=1.16, *p*=.341, η^2^p= 0,1.

### OSI results

For the Task-Oriented subscale, Mauchy’s test  χ^2^(2)=0.43, *p*=.81. For this subscale, no significant effects were found, neither for time, F(2,38)=0.04, p=.965, η2p=0,00, nor for the interaction with the group belonging, F(6, 38)=1.50, p=.204, η2p=0,19.

For the Logic subscale, Mauchy’s test  χ^2^(2)=2.23, *p*=.328. For this subscale, no significant effects were found neither for time, F(2,38)=1.29, p=.313, η2p=0,05, nor for the interaction with the group belonging, F(2,38)=1.92, p=.103, η2p=0,23. In spite of these results, contrasts revealed a significant interaction effect of the group between T1 and T2, F(3,19)=3.39, p=.039, η2p=0.35.

For the Mental Health subscale, Mauchy’s test  χ^2^(2)=1.35, *p*=.508. For this subscale, no significant effects were found, neither for time, F(2,38)=1.78, p=.183, η2p=0,09, nor for the interaction with the group belonging, F(6, 38)=0.88, p=.518, η2p=0,12.

For the Physical Health subscale, Mauchy’s test  χ^2^(2)=4.38, *p*=.112. For this subscale, no significant effects were found neither for time, F(2,38)=2.46, p=.099, η2p=0,12, nor for the interaction with the group belonging, F(2,38)=1.29, p=.284, η2p=0,17. In spite of these results, contrasts revealed a significant interaction between T1 and T2, F(1,19)=7.19, p=.015, η2p=0.28.

For the Social Support subscale,Mauchy’s test (χ^2^(2)=2.08, *p*=.35.

For the Home-Work Relation subscale, Mauchy’s test χ^2^(2)=0.37, *p*=.83.

For the Time subscale, Mauchy’s test χ^2^(2)=2.61, *p*=.271

For the Involvement subscale Mauchy’s test χ^2^(2)=0.54, *p*=.762

For the following subscales, no significant effects were found at any level: the Social Support subscale, did not show any significance neither for time, F(2, 38)=0.10, *p*=.903, η^2^p=0,01, nor for the interaction with the group belonging, F(6,38)=0.85, 1.37, *p*=.253, η^2^p=0,18; the same for  the Home-Work Relation subscale, time (F(2, 38)=0.65, *p*=.530) and interaction effect with group (F(6,38)=1.87, *p*=.112, η^2^p=0,23);  Time subscale, time (F(2, 38)=1.07, *p*=.353, η^2^p=0,05) and interaction effect with group (F(6,38)=0.34, *p*=.914, η^2^p=0,05); and Involvement subscale, time (F(2, 38)=0.14, *p*=.868, η^2^p=0,01) and interaction effect with group (F(6,38)=0.61, *p*=.724, η^2^p=0,09).

### PHQ-8 and GAD-7 results

For the PHQ-8 test, Mauchy’s test indicated that the assumption of sphericity had been violated for the main effect of time, χ^2^(5)=18.51, p=.002, therefore degrees of freedom were corrected using Greenhouse-Geisser estimates of sphericity (ε = .74). Despite this, no significant effects were found, neither for time, F(2.22, 68.69)=0.78, p=.47, η^2^p= 0,03, nor for the interaction with the group belonging, F(6.65, 68.69)=0.87, p=.53, η^2^p= 0,08.

For the GAD-7 test, Mauchy’s test with χ^2^(5)=3.41, *p*=.64 indicated that the assumption of sphericity had not been violated, therefore, corrections were not necessary. The results did not show any significant effect of the interaction between time and belonging group, F(9,93)=, *p*=.184, η^2^p=0,12; whereas, significant effect of the time on the considered variable was found, F(3,93)=2.96, *p*=.036, η^2^p=0,09. Repeated contrasts were performed to better understand this interaction, with no significant results. For this, simple contrasts were performed by comparing each measurement time with the baseline level; these contrasts showed a significant interaction when comparing T4 with T1, F(1,31)=7.11, *p*=.012, η^2^p= 0,19.

### Participants’ feedback

One-Way Anova was performed to assess differences between groups regarding evaluation of usefulness, ease and satisfaction with the treatment.

In terms of overall satisfaction, for which G1, G2 and G3 were considered, no statistical difference was found, F(2,31)= 0.61, *p*= .550, η^2^= 0.04, as well as for overall utility, F(2,31)=1.40, *p*= .261, η^2^= 0.08.

For measurements related to the use of TEO, for which G2 and G3 were considered, no significance was found, respectively for: easy of use, F(1,18)= 0.12, *p*= .738, η^2^= 0.01; usefulness, F(1,18)= 0.04, *p*= .846, η^2^= 0.00; personal usage, F(1,18)= 0.97, *p*= .338, η^2^= 0.05.
